# Temporal dynamics and tissue-specific variations of the blueberry phyllosphere mycobiome

**DOI:** 10.1093/hr/uhaf042

**Published:** 2025-02-12

**Authors:** Shay Lychen Szymanski, Timothy David Miles

**Affiliations:** Department of Plant, Soil and Microbial Sciences, Michigan State University, 1066 Bogue Street, East Lansing, MI, USA, 48824; Department of Plant, Soil and Microbial Sciences, Michigan State University, 1066 Bogue Street, East Lansing, MI, USA, 48824

## Abstract

Highbush blueberry (*Vaccinium corymbosum*) is an economically important fruit-bearing woody perennial. Despite the importance of microbial communities to plant health, the aboveground (phyllosphere) microbiome of blueberry is understudied. The phyllosphere is exposed to varying conditions throughout a growing season. The fruit undergoes extensive physiological change across a season from bud to fruit. This study aimed to provide a temporal characterization of the blueberry phyllosphere across a growing season and a characterization of specific tissues and phenological stages. Blueberry branches were harvested every other week across 2 years and two locations during the development process of the blueberry fruits. The internal transcribed spacer regions were amplified from DNA extracts and sequenced to perform amplicon-based characterization of the fungal microbiome across time and plant tissue. Fungal communities showed changes in α-diversity depending on the week of harvest and tissue type. Early in the season, α-diversity was high, but it decreased in midseason when flowers developed into fruit. Later in the season, as the fruit ripened, α-diversity increased again. The β-diversity of the community changed across time and tissue types during plant development. Notable members of the identified core microbiome were members of the genus *Alternaria*, *Peltaster*, and *Taphrina*, as well as the pathogenic taxa *Aureobasidium pullulans* and *Botrytis cinerea*. This research provides background for future experimentation of understanding the microbial composition in the blueberry phyllosphere in relation to the infection court of pathogens (e.g. *Colletotrichum fioriniae* and *B. cinerea*) and the temporal components of blueberry plant health and management.

## Introduction

Plants are host to diverse and robust microbial communities throughout the entirety of the plant, from seed to maturity [1]. At the extremes these microbes can be beneficial (e.g. nitrogen-fixing bacteria on roots and leaves) or detrimental (e.g. plant pathogens) [2, 3]. However, most taxa in the community are commensal, providing cryptic benefits or impacts that are not fully understood, although any of the above categories can shift to another depending on the context [4]. As such, characterizing the microbial communities of plants can provide insights into plant health and function [3].

The phyllosphere is composed of all aboveground portions of the plant, such as the stems, leaves, flowers, and fruits [5]. These different tissue types often differ in the composition of their microbial communities, particularly in the case of fungi [6]. While the community of the phyllosphere is mostly consistent with the rhizosphere and is driven by rhizosphere-mediated recruitment [7], studying the phyllosphere is beneficial for comprehensive characterization of the crop’s microbial community. The phyllosphere of a crop is subject to radical changes throughout a growing season capable of causing shifts to the microbial communities [8]. Some of these changes include daily temperature fluctuations, severe weather events, interactions with pollinators [9], and cultural and chemical disease management [10]. These community shifts may influence plant health [8]. Furthermore, in many cases the actual harvested product is of the phyllosphere.

Highbush blueberry (i.e. *Vaccinium corymbosum* L.) is a perennial woody fruit-bearing plant cultivated globally with a growing industry [11]. While economically important, the microbiome of blueberry is understudied, with few studies characterizing the rhizosphere [12, 13], and even fewer characterizing the fruits themselves [10]. Examination of the blueberry phyllosphere can provide a baseline understanding of what taxa are in which tissues, as well as understanding how these communities may change over the course of the season. This information could be helpful for informing management decisions and practices, such as pruning and fungicide applications [14].

**Fig. 1:**
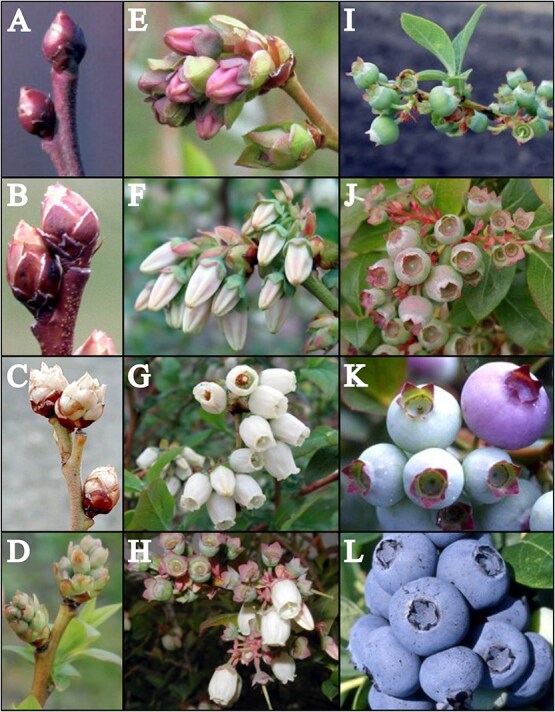
Growth stages of highbush blueberry fruits. Fruits start out as dormant tight buds (A) developed in the previous season. These buds swell (B) and eventually break (C), dropping scales. Tight bud clusters (D) develop and elongate (E). These buds lose color (F) as they further develop into flowers. Flowers enter bloom for a brief period (G), after which petals fall leaving exposed stamens (H). At this point, fruit begin to develop. Initially, fruit are small and tough with no distinct coloration (I) but continue to grow through the season (J). As the season progresses, fruits will begin to change color as anthocyanins accumulate (K) and fruit ripen. Eventually, fruits will be recognizably ripe (L). Fruits on the same bush and even cluster do not all reach full coloration at the same time. Photos adapted with permission from MSU Extension [26].

Ephemeral tissues such as buds and flowers undergo substantial changes in size, physiochemical properties, and nutrient content, facilitating dynamic changes in their microbial community. In apple, blossoms were observed to undergo microbial community succession over the course of weeks [15]. Flower microbial communities are further influenced by the pollinators they attract [9], which may in turn inform the microbial community of the later derived fruits [16]. Additionally, these tissues may produce compounds that influence microbial activity. In blueberry, floral extracts have been determined to induce appressorium formation of *Colletotrichum fioriniae* in a cultivar-specific manner [17].

Understanding the community dynamics of ephemeral tissues in blueberry is important because some of the most notable plant pathogens infect during bloom but do not always exhibit symptoms until later as fruit develop and ripen. In the USA, highbush blueberry production is primarily threatened by mummy berry, caused by *Monilinia vaccinii-corymbosi,* and postharvest fruit rots, namely *Alternaria* sp., *Botrytis cinerea*, and *C. fioriniae* [14]*. Monilinia vaccinii-corymbosi* infects at flowering and replaces developing ovaries, eventually producing mummified fruits [18]. *Botrytis cinerea* and *C. fioriniae* can infect flowers, forming initial infections and remaining quiescent throughout the growing season until fruit ripen, often only causing symptoms after harvest occurs. This can lead to substantial losses, in some cases up to 100% yield loss [19]. Little is known about the behavior of these pathogens during their dormant phases, so understanding their ecological context could prove valuable to the broader understanding of each pathosystem.

The phenological stage of bloom is a crucial period for pathogen management. While the fruit rot pathogens can be partly managed by cultural control measures such as pruning and planting certain cultivars, no cultivar is immune to these pathogens [20]. Therefore, management is primarily achieved using fungicides. However, the use of fungicides during bloom poses challenges for pollinator stewardship. While there are best practices that can be utilized to avoid harm to pollinators, there is desire for alternative treatment options [21]. Furthermore, there is mounting fungicide resistance in blueberry pathosystems [22, 23], with evidence suggesting that fungicide-resistant pathogens can become dominant members of the fruit microbiome when treated with fungicides [10]. Biological controls are less likely to be detrimental to pollinators but may also be less reliable in their efficacy [24]. Characterization of native microorganisms of the floral tissues and beyond may help identification of taxa with greater affinity for blueberries with disease-suppressive potential. Recent work has demonstrated that passaging phyllosphere microbial consortiums between successive growing periods can help facilitate selection of suppressive combinations of taxa [25], which may bring greater importance to this application of microbiome research in coming years.

This study examined the phyllospheric microbiome of ‘Bluecrop’ highbush blueberry plants in two plots in 2020 and a single plot in 2021. Samples were collected from May to August, capturing various tissues at each time point. It was expected that there would be a location-based difference between the two plots in 2020 as well as a year-based effect between samples collected in 2020 and 2021. The overall hypothesis was that the microbial communities would differ between the tissue types sampled (stem, leaf, bud, flower, green fruit, coloring fruit, and blue fruit, Fig. 1). Also, the bud, flower, and fruits would undergo rapid changes in these microbial communities within the season in comparison to the stems and leaves. Furthermore, we anticipate that the stem tissue will lack microbial diversity, while the flower and blue fruit will have the greatest diversity. Additionally, the taxa present in the flowering stage will inform the taxa present in the fruit stages.

## Results

### Microbial diversity is mediated by plant tissue

**Fig. 2:**
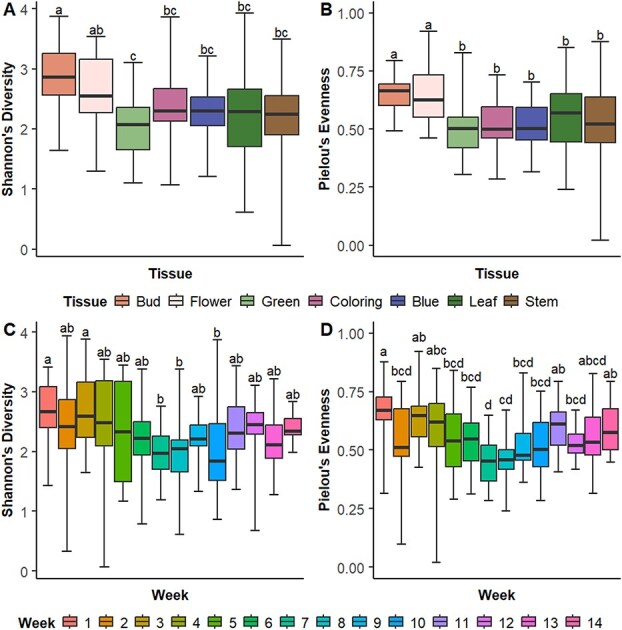
Differences in α-diversity measures by tissue type (A: Shannon’s index, B: Pielou’s evenness) and by week of sampling (C: Shannon’s index, D: Pielou’s evenness) among all samples

Among all samples, there were differences between some tissue types in terms of α-diversity. Bud and flower tissues had the highest diversity of operational taxonomical units (OTUs) in terms of both Shannon’s Diversity index and Pielou’s evenness (i.e. similarity of OTU abundances within a sample), while the green fruit had the lowest Shannon index (Fig. 2A).

**Fig. 3:**
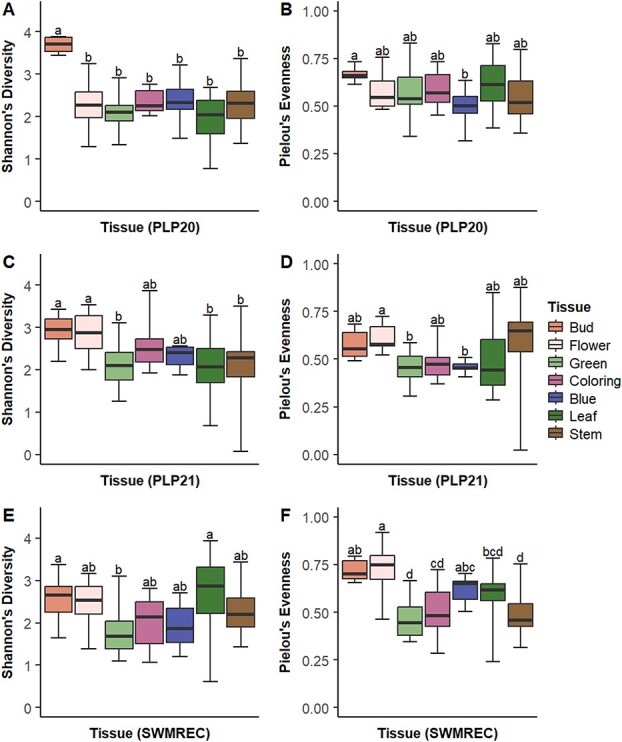
Differences in α-diversity measures (A, C, E: Shannon’s Diversity and B, D, F: Pielou’s evenness) by tissue type among samples from each site–year (A, B: PLP20, C, D: PLP21, and E, F: SWMREC).

Differences of α-diversity within tissues were identified within each site–year as well. For samples harvested from Michigan State University’s Plant Pathology Farms in 2020 (PLP20), bud tissue had the greatest diversity and evenness, but blue fruits had the least evenness (Fig. 3A and B). For samples harvested from the plant pathology farms in 2021 (PLP21), bud and flower tissues had the greatest diversity while flowers had the greatest evenness. Blue and green fruits had the least evenness (Fig. 3C and D). Within samples from the Southwest Michigan Research and Extension Center (SWMREC), diversity was greatest in the bud and leaf tissues and least in the green fruit. Flower tissue maintained the greatest evenness and green fruit tissue the lowest (Fig. 3E and F).

Between-sample diversity (β-diversity) demonstrated a gradient across the developmental stages of the blueberry fruit tissues. When examining all samples with nonmetric multidimensional scaling (NMDS) plots, there is overlap between each site–year, but there are distinct centers. Specifically, PLP20 appears between PLP21, which shares its location, and SWMREC, which shares its year. A similar gradient is present in the overlap between tissue types (Supplemental Fig. 1). When examining ordinations within each site–year, there is an overlap between different tissues. Ordinations without leaves and stems were also made to focus on the ephemeral reproductive tissues that are to become fruit (Supplemental Fig. 2). In these ordinations, there is a gradient between tissue types. Bud and flower tissues cluster together, while fruits cluster more closely with each other. Coloring fruit samples exist on a gradient between green and blue fruit samples, suggesting a gradual shift in microbial community composition as the fruit ripens.

### Microbial diversity varies across the growing season

**Fig. 4:**
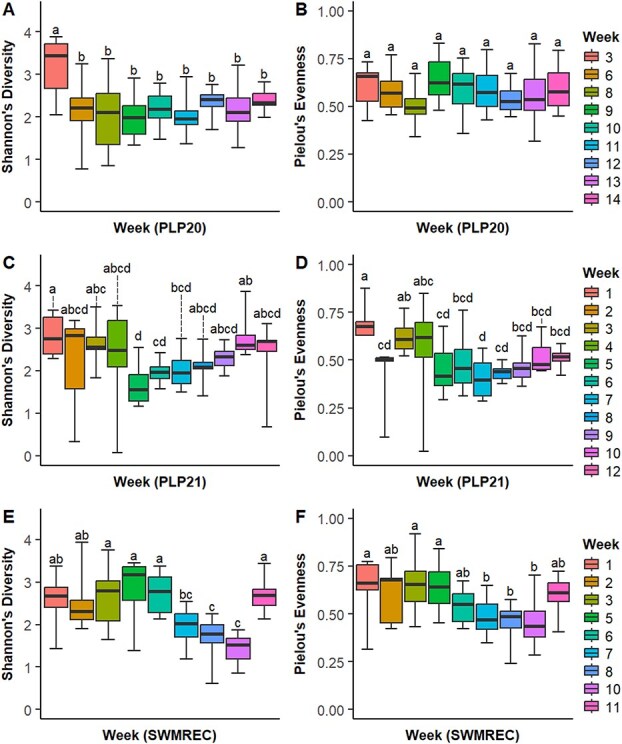
Differences in α-diversity measures (A, C, E: Shannon’s Diversity and B, D, F: Pielou’s evenness) by sampling week among samples from each site–year (A, B: PLP20, C, D: PLP21, and E, F: SWMREC)

α-Diversity relative to week demonstrated temporal differences in diversity. Weeks 1 and 3 had the highest Shannon index, while Weeks 7, 8, and 10 had the lowest. Week 1 had the greatest evenness and Week 7 the lowest (Fig. 2B). In PLP20, diversity was greatest in Week 3, with evenness having no significant differences (Fig. 4A and B). In PLP21, Week 1 had the greatest, and Week 5 the least diversity. Week 1 had the greatest evenness and Week 7 the least (Fig. 4C and D). In SWMREC, Weeks 3, 4, 5, and 11 had the greatest diversity, with Weeks 8 and 10 having the lowest. Evenness was greatest in Weeks 1, 3, and 5, and lowest in Weeks 7, 8, and 10 (Fig. 4E and F).

A permutational multivariate analysis of variance (PERMANOVA) demonstrated an influence of the week of sampling and the tissue sampled as well as their interaction on Bray–Curtis β-diversity. These analyses show a significant effect from tissue type, week, and their interaction. When restricting analysis to data from within each site–year, effects were greater (Table 1). There was a significant effect of sampling week and tissue type in each site–year as well as their interaction. β-Dispersion among weeks and tissues was also significant for each site–year except for β-dispersion by week in PLP20 and SWMREC (Table 1).

### Taxa demonstrate differential abundance between tissues and time

**Fig. 5:**
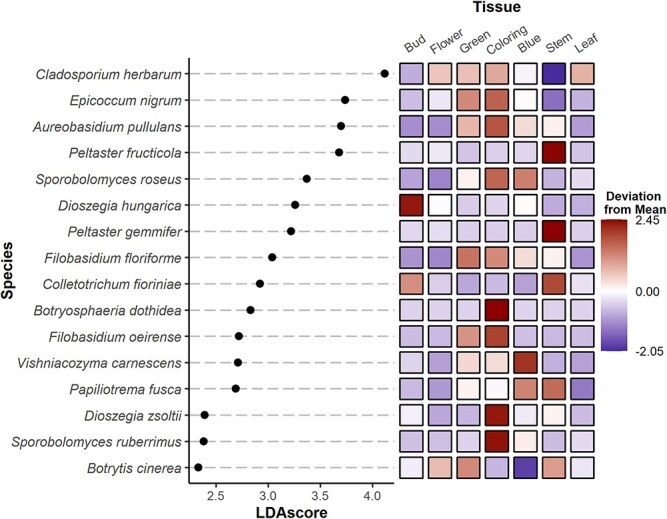
LefSe scores explaining the effect of tissues on species abundance. LDA scores are log-differential abundance scores. Coloration represents standard deviations that the mean abundance of each tissue is from the mean total abundance.

**Fig. 6:**
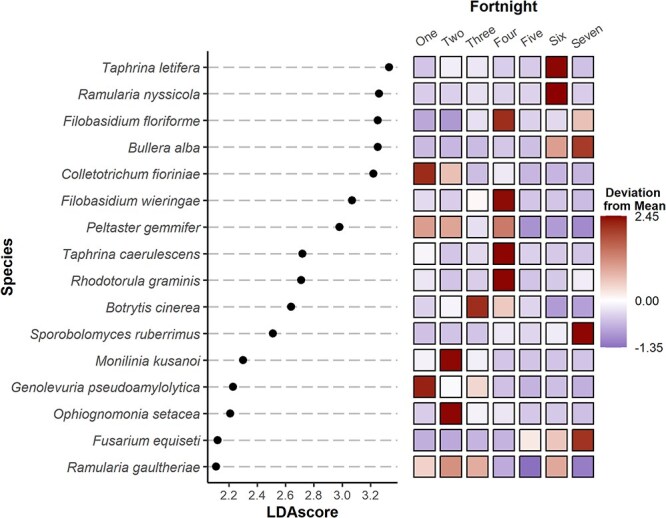
LefSe scores explaining the effect of fortnight (2-week sampling period) on species abundance. LDA scores are log-differential abundance scores. Coloration represents standard deviations that the mean abundance of each tissue is from the mean total abundance.

Linear discriminant analysis of effect size (LefSe) identified associations between taxa and specific tissues (Fig. 5) or specific 2-week periods (i.e. fortnights) (Fig. 6) of sampling. This was done at the species level between all samples. Among all samples, the highest LefSe scores were for *Cladosporium herbarum, Epicoccum nigrum, Aureobasidium pullulans,* and *Peltaster fructicola*, with *C. herbarum* showing less preference for the stem, *E. nigrum* and *A. pullulans* showing preference for green and coloring fruit*,* and *P. fructicola* showing preference for the stem. Other notable taxa are *C. fioriniae* showing preference for the stem and bud, and *B. cinerea* showing preference for the stem, flower, and green fruit.

The general growth form of each OTU based on the genus was examined by generating relative abundance plots of OTUs from each growth form category. Plots were generated from among all samples faceted by tissue type (Supplemental Fig. 3) and fortnight of sampling (Supplemental Fig. 4). These plots indicated bud and flower tissues have more filamentous fungi, while yeasts become more prevalent later in the season.

### Characterization of core microbiome and microbial interactions

A core microbiome across the season was identified for all samples and within each site–year (Supplemental Table 1). The core microbiome consisted of 65, 48, and 48 OTUs with respect to tissue, fortnight, and site–year, respectively, with 46 OTUs common to all three occupancy comparisons. A composite ranking for each core OTU by averaging the rank of OTUs from within each occupancy comparison. Core fungal taxa were all in the Dikarya (i.e. ascoymcetes and basidiomycetes) and were both yeasts and filamentous fungi. Some members of the core microbiome are potential rot pathogens, such as *Alternaria* sp.*, Cladosporium* sp., *E. nigrum*, and *B. cinerea. Alternaria* was found in most samples at a notable abundance. Other members of the core taxa were unexpected, namely four separate OTUs identified as *Taphrina* species.

Network analysis was performed using the sparse inverse covariance estimation for ecological association and statistical inference (SPIEC-EASI) method for each site–year and tissue type. Hub taxa were identified as those taxa with the greatest degree, betweenness centrality, and closeness centrality as determined by Network Analyzer in Cytoscape (Supplemental Table 2). Additionally, OTU interactions of interest were evaluated from these data (Supplemental Table 3). OTUs of interest were primarily hub taxa and taxa known to be pathogenic to blueberries.

**Table 1:** *R*  ^2^ values and β-dispersion of the influence of tissue type, week, and their interaction as determined by PERMANOVA for all samples and within each site–year.

***R*** ^**2**^ **of Factor**	**ALL**	**PLP20**	**PLP21**	**SWMREC**
Tissue	0.098	0.186	0.180	0.259
Week	0.095	0.116	0.153	0.167
Tissue × week	0.138	0.139	0.153	0.186
β-Dispersion (Tissue)	<0.001^*^	<0.001^*^	<0.001^*^	0.029^*^
β-Dispersion (Week)	<0.001^*^	0.097	0.047	0.421

^*^Significant β-dispersion values (α = 0.05).

## Discussion

Examining the blueberry phyllosphere mycobiome has revealed significant insights into the dynamics of fungal microbial communities. A consistent core microbiome, comprising 46 out of 65 identified core OTUs, was observed across all site–years, sampling times, and tissue types. This suggests a stable and resilient microbial community within the blueberry phyllosphere. A greater number of OTUs were considered core when ranked by tissue type rather than by site–year or fortnight, indicating that tissue-specific factors substantially influence microbial presence.

A variety of pathogenic taxa were among the core microbiome. The primary pathogenic species of *Alternaria* on blueberry are *Alternaria alternata*, *Alternaria arborescens*, and *Alternaria tenuissima* [27]*.* Discerning between these species is difficult with the ITS region alone, however, the most abundant *Alternaria* OTU has the greatest identity with an NCBI BLAST search with these species. The second most abundant *Alternaria* OTU has the highest identity with *Alternaria infectoria*, which is pathogenic on blueberry [27]. *Epicoccum nigrum* is also a pathogen of blueberries. However, *Epicoccum* sp. have previously been identified as a core member of the blueberry fruit microbiome [10] along with *Papiliotrema*, *Filobasidium*, and *Sporobolomyces* species. This is consistent with this study with exception to *Papiliotrema*, which while absent from the core microbiome was identified in various interactions during network analysis. *Filobasidium* and *Sporobolomyces* are both yeasts that are frequently isolated and sequenced in surveys of the phyllosphere [28] that have been identified as potential biological control agents due to their ability to rapidly colonize fruit surfaces and physically exclude pathogens from accessing wound sites [29]. These taxa likely provide a protective effect to the plant, particularly in the fruit where these yeasts were found to be more abundant.

The presence of *Taphrina* species in the core microbiome was unanticipated. This genus is typically host-specific and not often detected on asymptomatic plants, so its prevalence in the core when no *Taphrina* is known to infect blueberry is surprising. LefSe scores indicated an association with blue and coloring fruit, as well as flowers in the case of *Taphrina letifera*, which is typically associated with *Acer* plant species [30]. However, these OTUs shared only 96% identity with *T. letifera* and therefore could be a distinct undescribed *Taphrina* species. Furthermore, *Taphrina* sp. have been detected in amplicon-based studies in the phyllosphere of both highbush blueberries (*V. corymbosum*) [10] and wild blueberries (*Vaccinium myrtilus*) [31]. In Daghino [31] *Taphrina* was detected as a member of the core microbiome of *V. myrtilus,* being present throughout the whole plant. *Taphrina* species are capable of growing readily in their anamorphic yeast form in culture media, while their teleomorphic filamentous forms are those associated with pathology [32]. Despite this, *Taphrina* sp. are rarely isolated from asymptomatic tissues. It is possible that *Taphrina* species exist as saprobic epiphytic yeasts on various tissues with limited detriment to the host, supported by successful isolation of putative *Taphrina* sp. yeast states from nonhost species, floral nectar, and even plastic piping [33]. Together, these results indicate that the lifestyle of *Taphrina* species is more complex than previously understood and support the notion of a saprobic lifestyle among the anamorphs of the genus.

Across time points, α-diversity was the highest at the start of the season with a decline in diversity occurring in the middle of the season and a recovery in diversity near the end of the season. The lowest diversity and evenness occur overall in Week 7, the first week in which there is green fruit but no flowers. Furthermore, within the fruiting tissues, the highest diversity was observed in the buds and flowers, while the lowest diversity occurred within green fruit. Flowers as a peak in diversity could be related to their robust and accessible nutrients in the form of nectar [9], as well as a response by fungi to organic compounds produced by flowering tissue [17, 34]. It is known that compounds produced by blueberry flowers promote appressorium formation and secondary conidiation in *C. fioriniae* [17], so it is reasonable to suspect that other taxa may similarly be stimulated and promoted by flowering-related compounds. The high diversity in early season bud tissue was unanticipated and is more difficult to explain with a sparsity of literature discussing microbial communities of this tissue type. A study in apple has demonstrated that microbial populations associated with buds peaked during bud swell and supported abundant populations on both the exterior of the bud as well as interior bud scales, particularly those at the periphery [35]. Similarly, research in blueberry has indicated that the pathogen *Colletotrichum acutatum* overwinters primarily on the peripheral internal bud scales [36]. It is possible that the high diversity observed in bud tissue reflects a variety of taxa that overwintered from the prior season within the bud as well as taxa that colonized the exterior. The abundant surface area introduced by layered scales may also provide a diversity of niches available for colonization by varied microbes.

The development of green fruit coincides with a loss of diversity and this diversity recovers as the season progresses and fruits ripen. This may be related to the emergence of yeasts and dimorphic fungi in the fruits observed in this study. The lower diversity observed within this period could prove to be an ideal time for application of a compatible biological control agent as a less diverse microbial ecosystem may be more receptive to an introduced agent due to niche availability [37]. While the temporal patterns of diversity mirror the diversity of the tissues present, permutational analysis of variance indicates that the sampling week alone as well as its interaction with tissue type explains a sizable portion of the variation in diversity. This is likely due to a variety of factors such as variance in weather and changes in the physiochemistry of the fruits themselves as they develop.

The low evenness of green and blue fruits further reflects that this could be a prominent time for intervention with biological control agents. A lower evenness score reflects the dominance of a few taxa. There is a lower diversity of fungi that are readily able to grow in association with green fruit, with certain taxa becoming dominant as they are able to better and more rapidly colonize this tissue that is less facultative to most taxa. Our results indicate that *Filobasidium floriforme* and *A. pullulans* were observed to be highly associated with these tissues and are likely filling this role as early colonizers of these tissues. The rapid colonization by these yeasts allows them to exclude other taxa from effectively colonizing [38], with this control loosening as other niches may begin to open as fruit swell and change in physiochemical composition. However, other yeasts such as *Sporobolomyces* may be able to occupy these opening niches and come to dominate blue fruit, maintaining a low evenness despite an increase in diversity as fruits shift from green to blue fruit stages. *Sporobolomyces* sp. and *Aureobasidium* sp. have been identified to utilize killer toxins that can kill other yeasts as they compete for resources [39]. This competition among yeasts may explain why evenness remains low despite the increase in diversity.

A gradient was observed between tissue types that demonstrated a shift in community structure with fruit maturation and development. The dramatic shift between flowering and green fruit was counter to our expectation that floral taxa would be major drivers of fruit taxa. This shift could be driven by differences in nutrients or chemical composition of the blueberries. In *V. corymbosum* cv. ‘Bluecrop’, the same cultivar used in this study, there is increased respiration as fruits begin to gain color and transition from being immature green fruit [40]. This may be due to the metabolic activity needed to produce anthocyanin pigments that accumulate in the fruit skin during ripening, converted from the flavanols abundant in earlier green fruit stages [41]. Fungi associated with fruits may be promoted or suppressed by compounds along this spectrum. For example, *Colletotrichum acutatum* growth was promoted by anthocyanins from blueberry cultivars resistant and susceptible to anthracnose, but nonanthocyanin flavonoids suppressed growth, with greater suppression exhibited by flavonoids from the resistant cultivar ‘Elliott’ [42].

Ripening is also associated with an increase in sugars and an increase in pH [43]. These changes may be related to the increase in diversity observed with fruit ripening as a more neutral pH and greater abundance of simple sugars like sucrose should be able to support a broader range of microbial life. The sugar content of blueberry fruit has been positively correlated to anthracnose resistance [44], indicating that the increase in sugars can affect associated microbes. Other work has indicated that plant-associated sugars have been demonstrated to shape communities even in the rhizosphere [45], and more neutral pH has been identified to support greater bacterial diversity in phyllosphere microbiomes [46]. During this period, fruit become less firm [43], potentially making them more susceptible to invasion by mechanisms such as appressoria of phytopathogens [47]. A study in kiwifruit examined correlations between microbial taxa and various ripening parameters, finding that the abundance of 20 fungal taxa was significantly correlated with these parameters [48]. Among them, the abundance of *Sporobolomyces patagonicus* was positively associated with markers of more ripe fruits (i.e. soluble solids, ethylene content, and water-soluble pectin), which is consistent with the abundance of *Sporobolomyces* sp. associated with blueberries in this study. While not directly measured in this study, it is likely that blueberry fruits experience shifts in microbial community associated with these ripening-related changes.

*Colletotrichum fioriniae* was found to be associated with stems, flowers, and buds, while *B. cinerea* was associated with flowers, green fruit, and stems. *Colletotrichum fioriniae* was associated with Weeks 1 through 4, and *B. cinerea* with Weeks 5 through 8. The tissue and temporal associations of *C. fioriniae* are consistent with the known life cycle [19]. Sampling later in the season might have shown an increase in *C. fioriniae* in blue fruits due to the onset of ripe rot, which was not observed in this study. The association of *B. cinerea* with flowers aligns with its known life cycle and its role in botrytis blossom blight. These results are also consistent with previous work in wild blueberry (*Vaccinium myrtilis*), which identified *B. cinerea* as being preferential to flowers [31]. The connection with green fruit could result from sporulation on blighted flowers that persist into the early development of green fruit. Both pathogens can cause stem blights [49, 50], which may explain their affinity for stems.

Some yeast species (i.e. *Filobasidium* and *Sporobolomyces*) and mycorrhizal OTUs have specific tissue affinities, consistent with previous studies. Some OTUs linked with ericoid mycorrhizal formation were found in the phyllosphere as well. An OTU associated with *Hyaloscypha hepaticicola* was in the bud, stems, and green fruit at the PLP location in both years. Additionally, an OTU associated with *Serendipita* was found across all tissue types and site–years (data not shown). This is consistent with previous work in *Vaccinium myrtillus*, which similarly found ericoid mycorrhizal taxa in the tissues throughout the plant [31].

Examining hub taxa of different tissues and site–years, it is notable that hub taxa were frequently opportunistic plant pathogenic taxa, namely *Alternaria* sp. (Bud, Green, Coloring, SWMREC, PLP21) and *Epicoccum* sp. (Stem, Coloring, SWMREC, PLP21). In blue fruit, *C. fioriniae* was a hub taxon, suggesting that even before symptoms emerge that the pathogen begins to warp the fungal community. The host plants in this study were the cultivar Bluecrop which is susceptible to anthracnose [20], suggesting that *C. fioriniae* infection would follow an intracellular hemibiotrophic infection approach in which a haustorium is formed in an infected cell prior to initiating a necrotrophic phase [51]. The time of sampling and lack of symptoms in sampled fruits suggest that *C. fioriniae* may be acting as a hub even before initiating the more destructive necrotrophic phase. *Peltaster* species (i.e. *P. fructicola* and *Peltaster gemmifer)*, key members of the sooty-blotch and flyspeck (SBFS) complex in apples, were hub taxa in this study. In apples, *Peltaster* species, *Leptodontium elatius, Geastrumia polystigmatus*, *Stomiopeltis* spp*.*, and *Dissoconium* spp*.* cause a sooty-gray discoloration on the surface of apples [52]. Furthermore, *P. fructicola* appears in association with other *Peltaster* species and *Stomiopeltis* in blueberry without symptoms, although such discoloration would be more difficult to notice in smaller and darker fruits such as blueberry. However, blueberry plants may serve as an off-host reservoir for these fungi. The LefSe scores show *P. fructicola* and *P. gemmifer* being most strongly associated with stem and flower tissue, despite these taxa typically being associated with fruit epicuticular wax. These observations suggest that plant pathogens facilitate indirect effects to plant health in addition to their direct detriments.

Examining interactions between taxa revealed some relating to pathogens of interest. *Dioszegia* sp. were observed to be mildly antagonistic with *Alternaria* species, *C. fioriniae*, and *B. cinerea*. This suggests that these OTUs hold potential as biological control agents. Despite being identified as a hub taxon with potential suppressive activity in other cropping systems repeatedly [53, 54], not much work has been done to directly test the biological control capacity of *Dioszegia* species. Conversely, *P. fructicola* was repeatedly observed to be co-occurrent with pathogens such as *C. fioriniae*, *Alternaria* sp., *B. cinerea*, and *Taphrina* sp. This is consistent with its role as a key species in the SBFS complex [52]. Prior work has found *Peltaster*, *Alternaria, Botrytis,* and *Taphrina* species more prevalent in fungicide-treated fruits [10]. The occurrence of *Peltaster* species as both hub taxa and in positive associations with pathogens suggests that the epicuticular wax damage caused by these species may increase plant susceptibility to disease and that epicuticular wax could play a greater role in cultivar-based disease resistance. While *Sporobolomyces* sp. have been identified as potential biological control agents [55], in this study they were found to be positively associated with *Alternaria* species. Given the abundance and prevalence of both taxa and the flexibility of *Alternaria*’s ecological niche, this is not to say that *Sporobolomyces* sp. promote Alternaria fruit rot. However, it does complicate the implementation of the fungus as a biological control agent. Furthermore, recent work on passaging microbial communities of the phyllosphere through successive growth periods has shown promise at identifying microbial consortiums with disease-suppressive potential [25]. By repeating such work in blueberries, more promising candidates for disease suppression could be identified.

Distinguishing the microbial communities between the fruit skin and pulp could provide valuable insights, as prior research has shown these areas harbor different communities [10]. Investigating cultivar-based effects in blueberries merits further study. Resistance to various pests and pathogens, including postharvest rots like *C. fioriniae*, is correlated with the host genotype in highbush blueberry. Host genetics are known to influence microbiomes, including those in flowers. Recent research has attributed some of this variation to chemical traits such as the production of volatile organic compounds (VOCs) [34]. Resistance to *C. fioriniae* is related to the ability of blueberry floral extracts to induce conidiation [17], which could also affect other microbial residents. Therefore, further work linking host-plant traits such as VOC production, anthocyanin profile, pH, sugar content, and floral extract content to microbial communities could provide benefits to understanding microbial ecology and blueberry management strategies. Work of this nature would also benefit from performing a culture-based characterization in conjunction with culture-independent methods. Culture-based methods add further confidence to the results of a sequencing-based approach as well as provide workable cultures to directly examine observed microbe–microbe interactions, including potential biological control agents and disease-suppressive taxa.

## Conclusion

This work lays a foundation for further experimentation on blueberry microbial communities in the phyllosphere, identifying trends that warrant more targeted examination. More frequent and focused sampling of flowers during bloom could provide a more detailed understanding of the changes in fungal communities at this crucial stage for blueberry pathogens. Additionally, characterizing bacterial communities could enhance the understanding of the general microbial ecology, especially in flowers and fruits, which are sugar-rich, acidic environments that may support unique and diverse bacterial populations.

## Materials and methods

### Site description

In 2020, samples were collected from Michigan State University’s Plant Pathology Farms (PLP) located in East Lansing, MI, USA (42.6896, −84.4715), as well as from SWMREC located in Benton Harbor, MI, USA (42.0850, −86.3498). In 2021, samples were exclusively collected from the PLP location. Both locations of this study have conditions considered conducive to infection by fungal pathogens such as *C. fioriniae* [56]. SWMREC is located in southwest Michigan and experiences a potent lake effect that leads to increased snowfall, cloudier fall and winter seasons, and a moderation of temperature throughout the year. In comparison, PLP is located in south-central lower Michigan and experiences milder lake effect in the form of increased cloudiness in fall and winter [57]. PLP is USDA hardiness zone 6a, whereas SWMREC is 6b [58]. Both locations have soil that is sandy loam to loamy sand in composition, with PLP having well-draining soil and SWMREC having somewhat poorly drained soils [59].

### Sample collection

Samples were collected weekly to semiweekly in the years 2020 and 2021. Growths of approximately 5–7 inches were cut with ethanol-sterilized clippers to include all present tissue types. Cuttings were placed into sterile plastic bags and placed on ice until transferred to Michigan State University, East Lansing, MI, USA, and stored at −20°C until sample processing. A detailed listing of samples can be found at Supplementary Table 4.

### Sample processing

Samples were removed from −20°C and kept on dry ice prior while awaiting processing. In all instances, ~50–100 mg of sampled tissue was placed into sterile 1.5-ml microcentrifuge tubes in a sterilized biosafety cabinet. For bud samples, apical buds were sampled preferentially by removing the bud by cutting with a sterilized scalpel or clippers. Flowers were transferred to microcentrifuge tubes with sterilized forceps by grasping the pedicel. Fruits were bisected through the calyx and then again perpendicular to the calyx with sterilized scalpels. For coloring fruit, tissue from sectors with more advanced color development was selected. Leaves were separated with sterilized forceps and then cut repeatedly with sterilized scalpels. Leaf sections were taken from across the entire leaf, excluding the leaf petiole. Stem samples were taken by cutting the bottom inch with sterilized clippers and then cutting five to seven portions ~5 mm in length above this portion. Nodes were excluded from this portion. Samples were lyophilized overnight to remove moisture for more effective bead milling and transferred to 2-ml tubes with sterilized steel beads (2.4 mm) and processed at 300 Hz for 5 min in a bead mill prior to extraction. For nonstem samples this was sufficient to reduce samples to fine powder. Stem samples underwent an additional bead-milling step at 300 Hz for 5 min to be reduced to a similar state.

### DNA extraction

DNA was extracted from samples with an Omega BioTek Mag-Bind Plant DNA extraction kit following manufacturer protocols except for the addition of sterile polyvinylpyrrolidone (PVPP40) (final concentration 2% w/v) and dithiothreitol (final concentration 40 mM) to the lysis buffer [10]. Briefly, 500 μl of the modified lysis buffer was added to the lyophilized and pulverized samples and were vortexed before being incubated at 56°C for 30 min. Then, samples were centrifuged at 4000 × *g* for 10 min. Four hundred microliters of the cleared lysate was transferred to a sterile deep-well plate. Five microliters of RNase A was added to each sample and briefly vortexed to mix prior to incubation at room temperature for 10 min. Four hundred microliters of isopropanol and 15 μl of paramagnetic beads were added to each sample and vortexed to mix prior to incubation at room temperature for 5 min. The deep-well plate was placed on a magnetic separation device at room temperature until magnetic beads were cleared from solution. The cleared supernatant was aspirated and discarded before removing the plate from the magnetic separation device. Five hundred microliteres of wash buffer was used to resuspend the magnetic beads by pipetting up and down. The deep-well plate was returned to the magnetic separation device until magnetic beads were cleared from solution. This process is repeated three times with two different wash buffers. After the last wash buffer step, the magnetic beads were left on the magnetic separation device and allowed to air dry for 10 min. The deep-well plate was then removed from the magnetic separation device before resuspending the beads in 100 μl of elution buffer that had been preheated to 65°C by pipetting up and down. The deep-well plate was incubated at 65°C for 10 min. The deep-well plate was returned to the magnetic separation device until the magnetic beads were cleared from solution. The supernatant was transferred to a 96-well shallow plate and sealed with aluminum cover foil before storing at −20°C until further use.

### Amplicon generation and sequencing

A three-step polymerase chain reaction (PCR) protocol was followed to generate amplicons [60] with the base primers ITS1f [61] and ITS4 [62]. To prevent amplification of the host ITS region, a peptide nucleic acid (PNA) clamp was designed against *V. corymbosum* [63] and utilized in steps one and two of the three-step PCR protocol (N′- TGGCCCAGCTGCCTCG – C′). Samples were normalized to concentrations of ~1–2 ng/μl with a silica plate (Applied Biosystems) and purified with paramagnetic beads (Mag-Bio Genomics). Amplicon size and quality was verified by gel electrophoresis with a 2% w/v agarose in TAE gel. Sequencing was performed with 1 μl of purified sample with Illumina Mi-Seq v3 300-bp paired-end sequencing at the Michigan State University genomics core.

### Bioinformatics

Sequencing data were processed using the Cecilia pipeline [64]. Briefly, reads were quality assessed with FASTQC [65]. Adapters and primers were removed from reads with Cutadapt [66]. Due to the inability to join forward and reverse reads, OTUs were generated exclusively with forward reads. Before processing, 39 921 056 reads were generated from sequencing. Forward reads were quality assessed at various lengths with USEARCH [67] and then trimmed to a consistent 225-bp length. This length was chosen for the number of reads retained when filtering out reads with an expected error rate of ≥1% as well as information provided by each read. After filtering, 38 565 751 reads remained. Reads were dereplicated and OTUs generated using UPARSE [68] at 99% clustering. Taxonomic assignment of generated OTUs was performed initially by CONSTAX v2 [69]. Taxa unable to be resolved at the phylum level were further examined utilizing NCBI-Blast to identify potential contaminants or well-documented taxa not represented in curated databases. The FungalTraits database [70] was utilized to provide a characterization of lifestyle aspects based on the genus of each OTU.

### Statistical analysis

All statistical analysis was performed in R v 4.2.3 [71]. Prior to analysis, contaminants were identified and removed with the package *microDecon* [72]. A total of 2 121 705 reads were removed this way. Any taxa associated with plant or metazoan species were also removed, with a final N reads used in further analysis. α-Diversity analysis was performed with the package *vegan* [73] with the function ‘diversity’ for Shannon’s diversity and ‘specnumber’ to obtain species number. Pielou’s evenness was defined as the Shannon’s index divided by the log of the result of ‘specnumber’ [73]. Analysis of variance (ANOVA) was performed to determine differences between means with the base R function ‘aov’ (*P <* .05). Differences between groups were assessed with Tukey’s honest significant difference with the function ‘HSD.test’ in the package *agricolae* (*P <* .05, groups = TRUE, unbalanced = TRUE) [74]. β-Diversity analysis was performed as in Szymanski et al 2023 [10]. Briefly, OTUs with <10 reads were removed to reduce noise from sequencing errors. Samples were normalized by cumulative sum scaling with ‘cumNorm’ from the *metagenomeSeq* package [75]. Then, the function ‘adonis2’ from *vegan* was used to perform a PERMANOVA to assess effects of variables (i.e. week, tissue) on Bray–Curtis dissimilarity with 9999 permutations. To assess β-dispersion, the function ‘vegdist’ was used to acquire distances between observations to be interpreted by ‘betadisper’. The results from ‘betadisper’ were evaluated with the ‘anova’ function from the core R *stats* package [71]. Ordinations were made with the same normalized OTU tables using the function ‘ordinate’ (method = ‘NMDS’, distance = ‘bray’) in *phyloseq* [76]. Core membership was defined as taxa whose addition to the population contributed a ≥2% increase to Bray–Curtis diversity when added in order of occupancy with respect to tissue, fortnight, and when examining all samples, site–year using a modified script from Shade and Stopnisek [77].

Network analysis was performed with the function ‘*spiec.easi*’ (method = ‘mb’, lambda.min.ratio = 1e-5, nlamda = 30) using the package Spiec-Easi [78]. OTUs occurring in <10% of samples of each corresponding factor and had a total abundance of ≤10 reads were filtered prior to analysis with total counts retained. Cytoscape version 3.10 was additionally utilized to visualize networks [79] and the Analyze Network function was utilized to help identify hub taxa [80]. LefSe analysis was performed with the package MicrobiomeAnalystR [81] with the function ‘PerformLefseAnal’ (fdr cut-off = 0.05, LDA cut-off = 2.0). All plots were generated with the package *ggplot2* [82].

## Supplementary Material

Web_Material_uhaf042

## Data Availability

Raw sequencing data generated for this study can be found in the NCBI SRA archive as Bioproject PRJNA1161654. Code is available at https://github.com/szymanskishay/blueberrytimecourse/
