# Supplementary figures and images for: Temporal dynamics and tissue-specific variations of the blueberry phyllosphere mycobiome
Source: Hortic Res. 2025 Feb 12;12(5):uhaf042. doi: 10.1093/hr/uhaf042 (PMC11997430; doi:10.1093/hr/uhaf042)

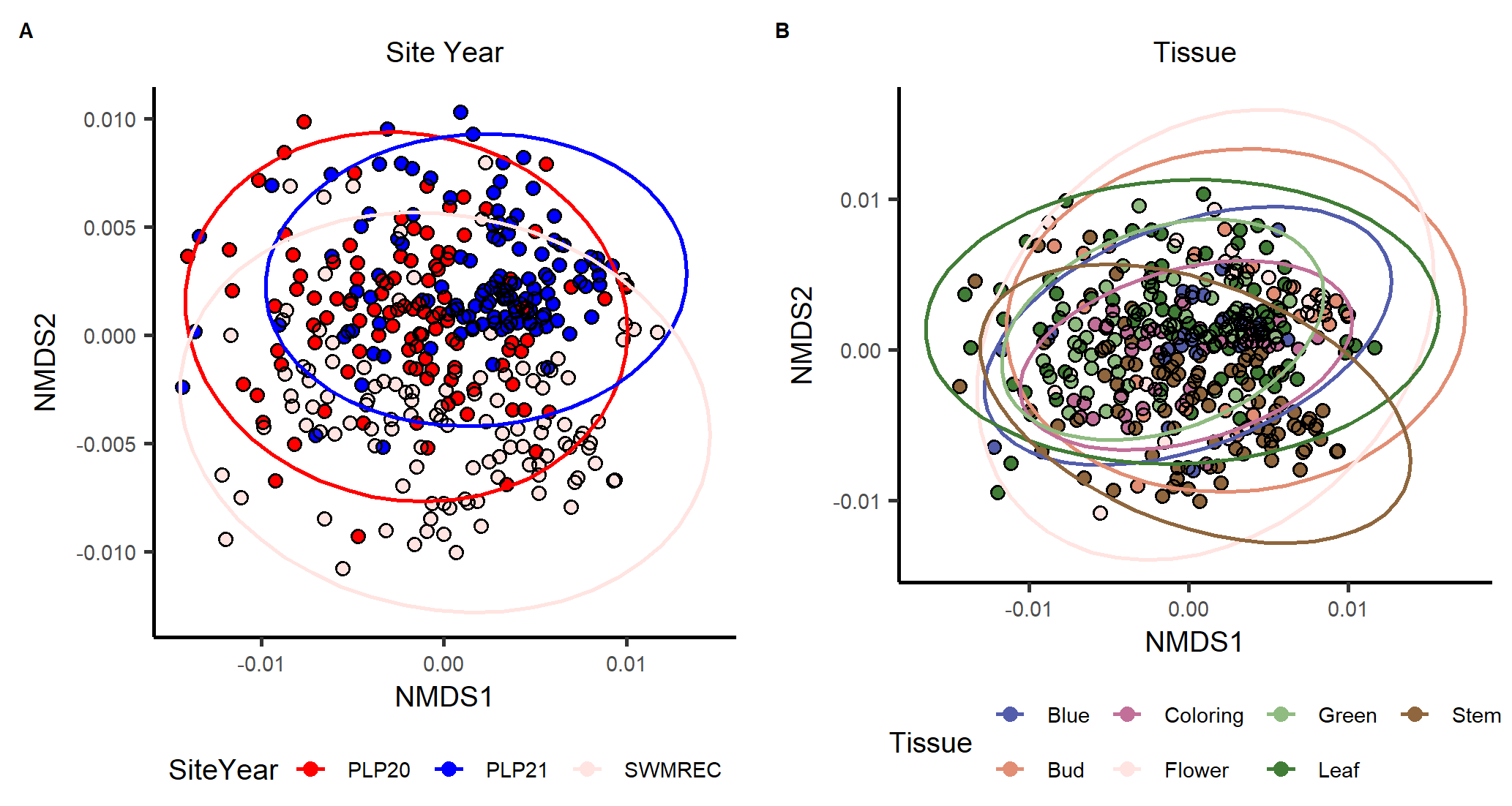

Supplement: Web_Material_uhaf042 [file web_material_uhaf042.zip › supplementalfigure1.tiff]

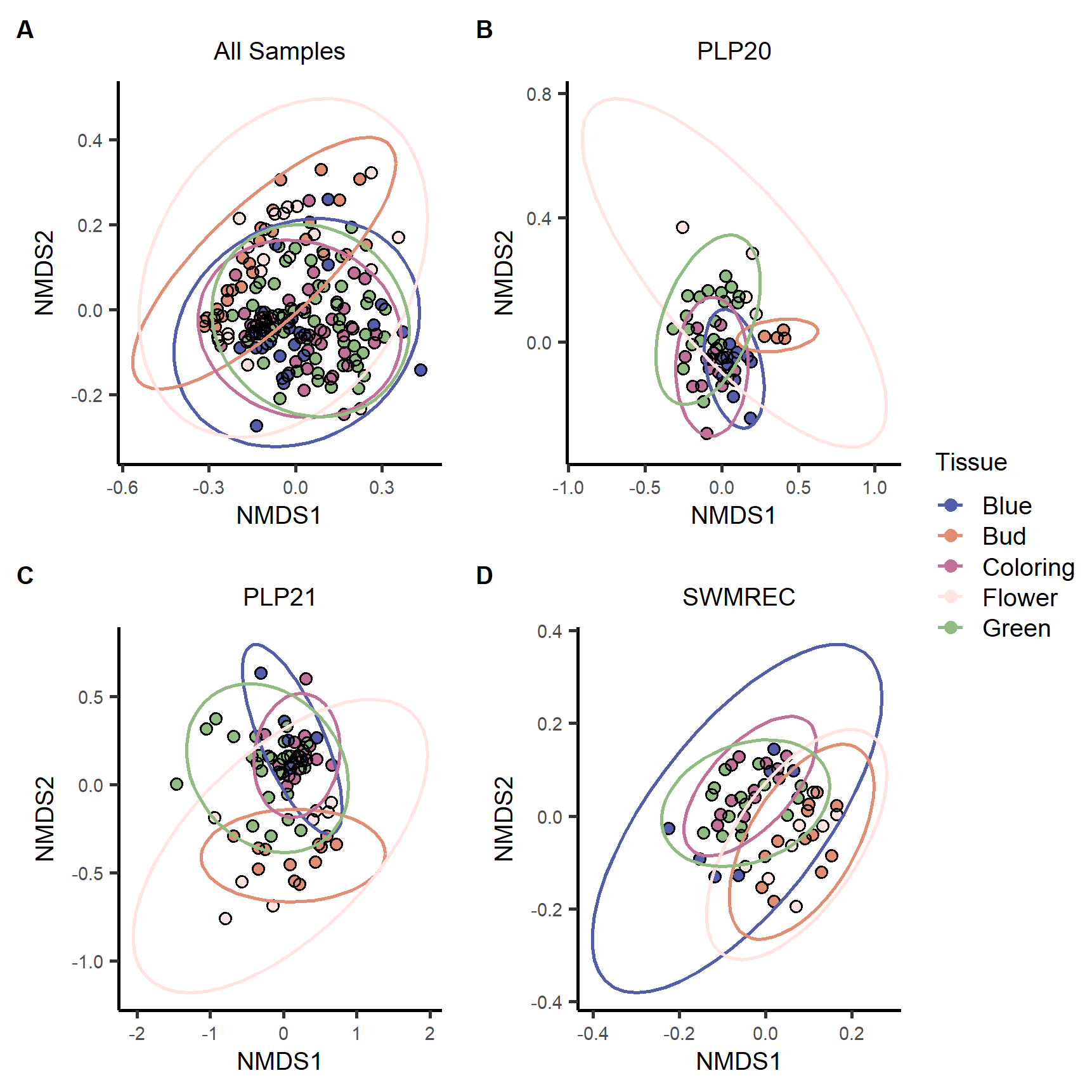

Supplement: Web_Material_uhaf042 [file web_material_uhaf042.zip › supplementalfigure2.tiff]

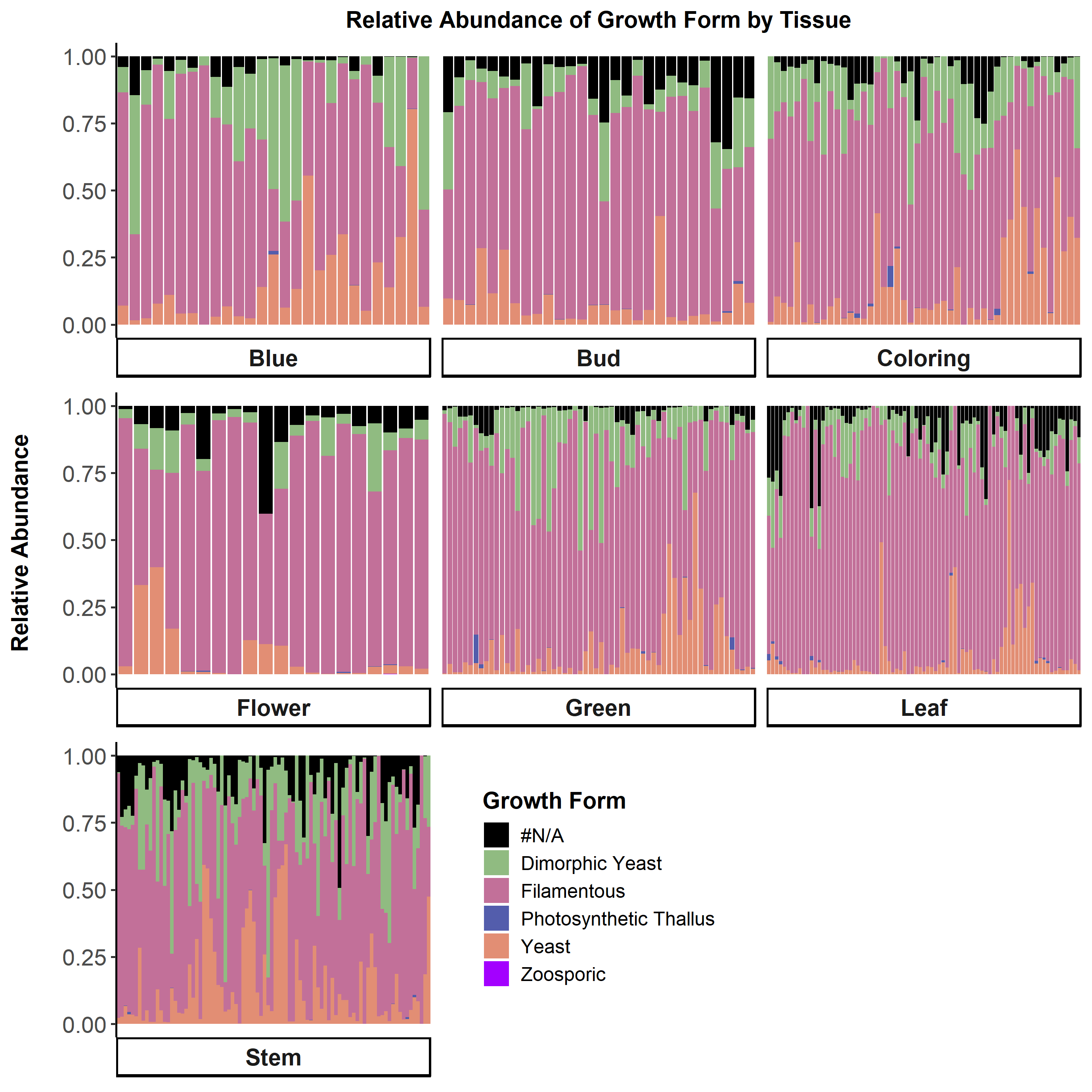

Supplement: Web_Material_uhaf042 [file web_material_uhaf042.zip › supplementalfigure3.tiff]

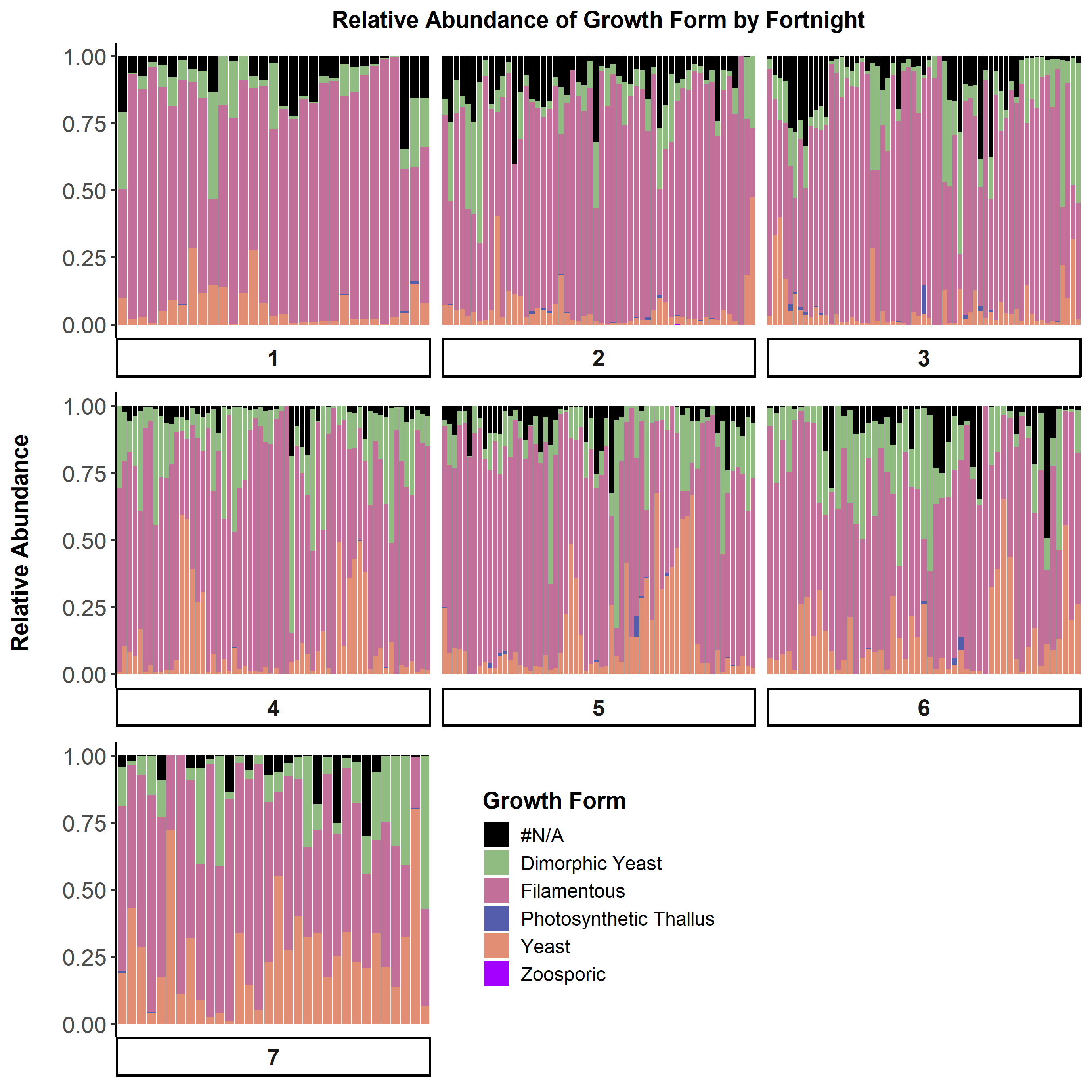

Supplement: Web_Material_uhaf042 [file web_material_uhaf042.zip › supplementalfigure4.tiff]
